# Provision of infertility care for the underserved in reproductive endocrinology and infertility practices associated with obstetrics and gynecology residency training programs in the United States

**DOI:** 10.1016/j.xfre.2021.11.002

**Published:** 2021-11-05

**Authors:** Tia Jackson-Bey, Holly Mehr, Jacqueline R. Ho, Molly M. Quinn, Lusine Aghajanova, Michelle Vu, Christopher N. Herndon

**Affiliations:** a Reproductive Medicine Associates of New York, New York, New York; b Department of Obstetrics and Gynecology and Reproductive Science, Division of Reproductive Endocrinology and Infertility, Mount Sinai School of Medicine, New York, New York; c University of California, Los Angeles, Los Angeles, California; d University of Southern California, Los Angeles, California; e Stanford University School of Medicine, Stanford, California; f University of Rochester Medical Center, Rochester, New York; g University of Washington, Seattle, Washington

**Keywords:** Infertility, access to care, obstetrics and gynecology residency, barriers to care, disparities

## Abstract

**Objective:**

To survey practice patterns designed to increase access to infertility care and evaluate the exposure of obstetrics and gynecology residents to infertility care for the underserved.

**Design:**

Cross-sectional.

**Setting:**

Reproductive endocrinology and infertility (REI) practices associated with Accreditation Council for Graduate Medical Education-accredited obstetrics and gynecology residency training programs.

**Patient(s):**

None.

**Intervention(s):**

Questionnaire survey.

**Main Outcome Measure(s):**

Presence of clinical programs designed to improve access to REI care, resident involvement in such programs, and perceived barriers to expanding access to care.

**Result(s):**

Clinical initiatives to expand access included discounted infertility services (38%, n = 30), utilization of a low-cost in vitro fertilization (IVF) program (28%, n = 22), and utilization of a resident- and/or fellow-staffed clinic to provide infertility care (39%, n = 31). The most commonly discounted infertility services were IVF (73%, n = 22), clinical consultation (70%, n = 21), and intrauterine insemination (53%, n = 16). The provision of discounted prices was correlated with the increasing practice size (odds ratio [OR], 2.29; 95% confidence interval [CI], 1.23–4.24) and number of assisted reproductive technology cycles performed annually (OR, 3.65; 95% CI, 1.48–9.02). Academic REI practices (OR, 3.6; 95% CI, 0.98–13.25) were more likely to have a low-cost IVF program. Less than half of obstetrics and gynecology residency programs (39%, n = 31) had an associated REI clinic in which obstetrics and gynecology residents provide direct infertility care to the medically underserved. Frequency and services offered in trainee clinics varied. Multiple barriers to expanding access to care were reported.

**Conclusion(s):**

Reproductive endocrinology and infertility practices associated with obstetrics and gynecology residency programs utilize a diverse range of approaches to provide infertility care to the underserved in the backdrop of considerable challenges and barriers, but significant gaps persist.

**Discuss:** You can discuss this article with its authors and other readers at https://www.fertstertdialog.com/posts/xfre-d-21-00117

In 2009, the US Department of Health and Human Services reported that only 24% of the infertility needs in the US population were being met (1, 2). Over a decade later, these disparities persist and are further magnified among minority and immigrant communities (3). The cost of evaluation and treatment is the single largest barrier to accessing fertility treatment in the United States (4). Infertility treatments including in vitro fertilization (IVF) are not covered by most private, state, or public health insurance plans (5). The median price of a cycle of IVF is generally estimated to be $19,200 in the United States, with higher expenditures for successful live birth because patients may require more than 1 treatment cycle (6). While less-often cited, diagnostic evaluation and non-IVF fertility treatments are also cost-prohibitive for many, particularly those from lower-income strata. Multiple studies confirm the presence of wide inequities in access to care, with the majority of individuals who access infertility services being White, highly educated, and wealthy, despite infertility being more prevalent among non-White groups (7, 8, 9). In 2015, the American Society for Reproductive Medicine (ASRM) launched the Access to Care Initiative to address the wide disparities and inequities that exist in the United States and globally (10).

Residency and fellowship training programs at academic medical centers, community-based hospitals, and military medical centers throughout the United States often provide access to health services to underserved and marginalized communities. In this capacity, training centers may play a significant, and potentially underutilized, role in extending access to and the direct provision of infertility care for populations of need (11). In addition, engaging obstetrics and gynecology residents and reproductive endocrinology and infertility (REI) fellows in the provision of care can potentially lower health care costs, lessen current gaps in exposure and general knowledge of infertility, and better train graduates to provide quality and cost-effective infertility care in their future practices, which may include underserved areas or populations. Outside of a few published reports of specific clinical programs in the setting of training programs set up to expand infertility care to underserved patients (11, 12), little is known about what methods REI practices employ to expand access at teaching institutions across the country. Our study aimed to characterize the provision of infertility care to the underserved by US-based REI practices affiliated with the Accreditation Council for Graduate Medical Education (ACGME)-accredited obstetrics and gynecology residency programs, understand how residents are exposed to the care of patients with infertility in lower-resource settings, and determine the barriers encountered in expanding care in these settings through an anonymous survey.

## Materials and methods

### Survey Development

The survey was created and managed using Google Forms, an online professional survey platform, which encrypted the responses and identities of the survey respondents. The survey was pilot tested by reproductive endocrinologists from 3 academic assisted reproductive technology (ART) centers. The language of the survey was designed for either reproductive endocrinology or obstetrics and gynecology generalist physicians. A link to the anonymous survey was sent electronically to participants in April 2019.

The survey, provided in the Supplemental Material, collected information on demographics and also asked respondents about the strategies they utilize for provision of fertility care to underserved populations and their reported barriers in expanding access to care. Question types included a combination of single best answer, multiple choice, select all that apply and free text input. A response to every question was required for completion of the survey, but skip logic was used based on answers to specific questions. Thus, the participants answered between 15 and 28 questions. All questions had an “I don’t know” or “other” answer choice with a write-in option.

### Collecting Contact Information

Contact information for the clinician responsible for resident education in REI was not available for each obstetrics and gynecology residency prior to this study. We created a database of physician contacts through a 2-step process. First, using the publicly available FREIDA website (13), the name and email contact for each residency program coordinator and residency director at all 287 ACGME-accredited obstetrics and gynecology residency programs were obtained and maintained in a secure database. Each program coordinator was emailed up to 3 times to request contact information for “the physician or administrator who acts as the main point-person for obstetrics and gynecology resident training in REI.” Then, the provided REI physician contact information was compiled into a secure database.

### Recruiting Participants

An email introducing the project and providing a link to the anonymous electronic survey was sent to the REI resident education contact at each institution. The survey was sent at regular intervals 3 times from April 16 to April 28, 2019. No incentive for completing the survey was given.

### Statistical Analysis

Descriptive statistics were used to analyze survey responses. Logistic regression via Stata software was performed to identify factors associated with discounted fertility services, low-cost IVF program, and a trainee-run REI clinic for providing care in lower-resource settings. All data were coded to be categorically analyzed via logistic regression. The Fisher’s exact and χ^2^ test were used to evaluate the relationship between barriers to care in mandated and nonmandated states. A *P* value of <.05 was considered statistically significant. Geographic regions were determined according to the US Census Bureau Regions and Division.

The survey did not collect any personal health information. The study was considered exempt by the Institutional Review Board. Only data from completed surveys were included in the analysis.

## Results

Contact information for residency program coordinators was available for 276 of the 287 (96.2%) ACGME-accredited obstetrics and gynecology residency programs; 6 were excluded; of those excluded 3 had no contact email for the clinician responsible for resident REI education, and 3 programs replied with a lack of an REI rotation for the residents. Surveys were sent via email to physician REI resident education contacts affiliated with ACGME-accredited obstetrics and gynecology residency programs. The overall survey response rate was 30% (80/270).

### Demographics

The demographics of programs are summarized in Table 1. Responses were received from programs in 31 states and were equally distributed across all geographic regions. The average obstetrics and gynecology residency size of responding programs was 6.1 graduating residents per year (3–20 residents). Residents spent an average of 7.2 weeks (2–12 weeks) rotating through REI during a 4-year residency. Thirty-eight percent (n = 30) of programs had an affiliated REI fellowship.

**Table 1:** Demographics of REI practices affiliated with obstetrics and gynecology residency programs.

Demographics	n (%)
**Geographic region**	
Northeast	17 (21)
South	26 (33)
Midwest	23 (29)
West	13 (16)
n = 79	
**No. of providers**	
1–2	16 (20)
3–5	32 (40)
≥6	25 (31)
n = 71	
**Residency size**	
Small (<7)	41 (52)
Large (≥7)	38 (48)
n = 79	
**Weeks of REI exposure over 4-year residency**	
2–4	23 (29)
5–7	19 (24)
8–10	31 (40)
>10	10 (13)
n = 78	
**Fellowship**	
Yes	30 (38%)
No	50 (62%)
n = 80	
**Practice type**	
Academic	33 (41)
Private	19 (24)
Hybrid	21 (26)
Military	3 (4)
Other	4 (5)
n = 80	
**Offer IVF**	
Yes	70 (88)
No	10 (12)
n = 80	
**Cycles per year**	
<200	18 (26)
201–1000	38 (54)
>1000	14 (20)
n = 70	
**Embryology laboratory**	
On site	55 (79)
Off site	15 (21)
n = 70	
**Percent of patients unable to afford care**	
<10%	9 (11)
10%–25%	27 (34)
26%–50%	15 (19)
>50%	11 (14)
I don’t know	18 (23)
n = 80	
**Mandated state**	
Yes	16 (20)
No	63 (80)
n = 79	
**Oncofertility services**	
Yes	58 (83)
No	12 (17)
n = 70	

*Note:* IVF = in vitro fertilization; REI = reproductive endocrinology and infertility.

**Supplemental Figure 1:**
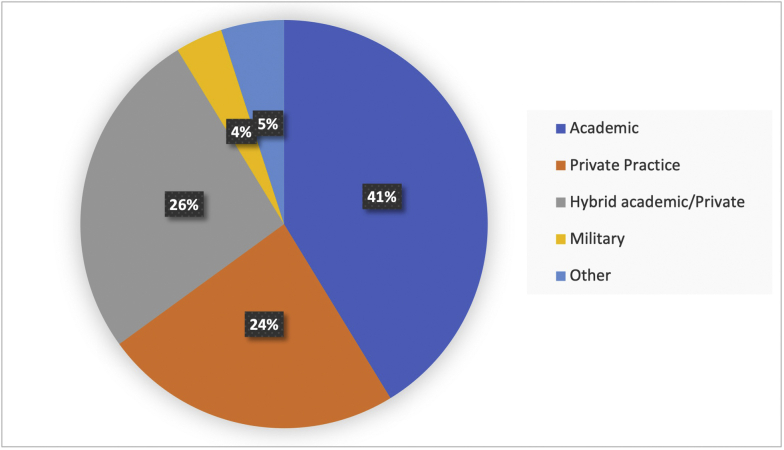
Reproductive endocrinology and infertility practice type of survey respondents (n = 80).

In regard to practice size, 20% (n = 16) of practices had 1–2 REI providers, 40% (n = 32) had 3–5 providers, and 31% (n = 25) had 6 or more providers. Of the respondents, 41% (n = 33) of REI practices associated with obstetrics and gynecology residency programs identified as academic, 24% (n = 19) private practice, 26% (n = 21) hybrid academic/private, 4% (n = 3) military practices, and 5% (n = 4) other (Supplemental Fig. 1, available online). Eighty-eight percent (n = 70) of practices offered IVF, and of those, 79% (n = 55) reported using an onsite embryology laboratory. The number of ART cycles per year was also equally distributed with 26% (n = 19) performing <200 cycles, 33% (n = 24) performing 201–500 cycles, 22% (n = 9) performing 501–100 cycles, and finally 19% (n = 14) performing >1,000 cycles (Table 1).

Fifty-six percent (45/80) of respondents reported at least 1 clinical practice or program to expand access to care to lower-income patients who are medically underserved or unable to afford infertility services. Respondents most reported offering discounted infertility services in their clinic (38%, n = 30), utilization of a low-cost IVF program (28%, n = 22), and utilization of a resident- and/or fellow-staffed clinic to provide infertility care (39%, n = 31).

### Resident and/or Fellow REI Clinic for Underserved Communities

Less than half (39%, n = 31) of responding obstetrics and gynecology residency programs had an associated REI clinic in which obstetrics and gynecology residents provide direct infertility care to populations who are medically underserved or unable to afford infertility services. Of those programs that had a resident-run clinic (Table 2), most clinics were held either at a resident GYN clinic (52%, n = 16) at their primary institution or at a county/public medical center (39%, n = 12). These trainee-staffed clinics were offered daily in 3 (10%) of 31 programs, weekly in 15 (45%), and 1 or 2 times monthly in 11 (35%) and at differing times on the basis of resident REI rotations at 2 clinics. The trainee clinics were staffed by a variety of providers; 48% (n = 15) were staffed by a resident and attending, 39% (n = 12) were staffed by a resident, fellow, and attending, and 13% (n = 4) were staffed by a resident with the fellow staffing as an attending. Twenty-one (68%) of the 31 programs with trainee-staffed clinics had no other clinical program to expand access to care.

**Table 2:** Characteristics of infertility clinic with trainee providers.

Characteristics	n (%)
**Trainee clinic**	
Yes	31/80 (39)
No	49/80 (61)
**Trainee clinic staff**	
Resident and attending	15/31 (48)
Resident and fellow as attending	4/31 (13)
Fellow and attending	0
Resident, fellow, and attending clinic	12/31 (39)
**Trainee clinic location**	
County hospital-based clinic	13/31(42)
IVF center	1/31 (3)
Resident obstetrics and gynecology clinic	16/31 (52)
Other	1/31 (3)
**Trainee clinic frequency**	
Daily	3/31 (10)
Weekly	15/31 (48)
Twice a month	6/31 (19)
Monthly	5/31 (16)
Other	2/31 (6)
**Services offered in clinic**	
Consultation	31/31 (100)
Hysterosalpingogram	20/31 (65)
Saline infusion sonogram	26/31 (84)
Pelvic ultrasound	27/31 (87)
Semen analysis	14/31 (45)
Clomiphene ovulation induction	26 (84)
Gonadotropin ovulation induction	5 (16)
Sperm preparation	3 (10)
Intrauterine insemination	6 (19)
Oocyte cryopreservation	3 (10)
In vitro fertilization	3 (10)
Intravaginal embryo culture	1 (3)
Donor in vitro fertilization	2 (6)
Tubal reversal surgery	6 (19)
Laparoscopy for tubal disease	23 (74)
Tubal canalization	8 (26)
Fertility care for HIV serodiscordant couples	3 (10)
Medical treatment of endocrinopathies	27 (87)
Endometriosis management	26 (84)
Uterine anomalies and disorders of sexual differentiation	21 (68)

*Note:* HIV = human immunodeficiency virus; IVF = in vitro fertilization.

**Supplemental Figure 2:**
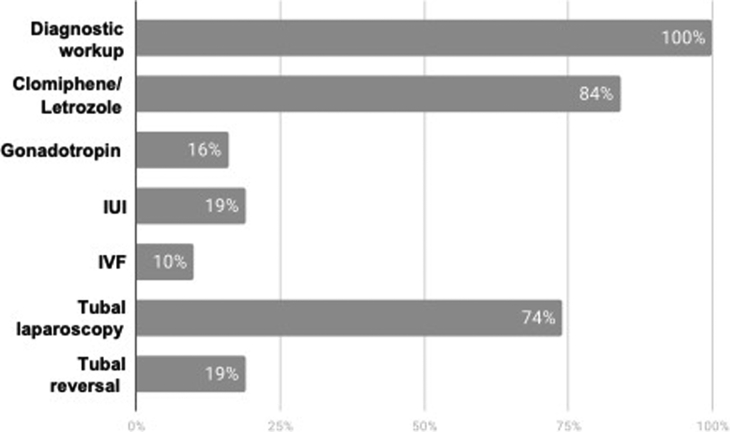
Clinical services offered in trainee reproductive endocrinology and infertility clinics (n = 31). IVF = in vitro fertilization; IUI = intrauterine insemination.

Clinical services offered in trainee-staffed REI clinics also varied: 100% (n = 31) offered some form of diagnostic evaluation, 84% (n = 26) provided treatment with clomiphene/letrozole, 16% (n = 5) provided treatment with gonadotropins, 19% (n = 6) provided treatment with intrauterine insemination, and 10% (n = 3) provided treatment with IVF. Seventy-four percent (n = 23) performed laparoscopy for tubal disease, and 19% (n = 6) offered tubal reversal surgery (Supplemental Fig. 2 and Table 2).

Of the following, no factor was significantly predictive of the presence of a trainee clinic: size of residency program (*P*=.18), REI practice setting (academic/hybrid/private) (*P*=.17), size of REI practice (*P*=.33), geographic region (*P*=.57), location in an IVF insurance mandated state (*P*=.21), or presence of an affiliated REI fellowship (*P*=.13) (Table 3).

**Table 3:** Predictors for infertility care provision for the underserved.

	Offer discounted fertility services n = 30 OR (95% CI) *P* value	Low-cost IVF program n = 22 OR (95% CI) *P* value	Resident or fellow clinic n = 31 OR (95% CI) *P* value
Geographic region	0.76 (0.47–1.24) *P*=.28	0.86 (0.53–1.41) *P*=.56	0.88 (0.57–1.36) *P*=.57
Practice size	**2.29 (1.23–4.24)** ***P*=.01**	1.46 (0.81–2.60) *P*=.21	1.28 (0.78– 2.11) *P*=.33
Residency size	0.92 (0.58–1.47) *P*=.73	0.51 (0.5–5.6) *P*=.68	1.36 (0.87–2.12) *P*=.18
Private vs. hybrid/academic	1.13 (0.34–3.71) *P*=.84	**3.6 (0.98–13.25)** ***P*=.05**	0.45 (0.14–1.41) *P*=.17
REI fellowship	0.875 (0.33–2.33) *P*=.79	0.49 (0.16–1.42) *P*=.19	2.06 (0.81–5.23) *P*=.13
Cycles per year	**3.65 (1.48–9.02)** ***P*=.005**	1.01 (0.45–2.23) *P*=095	1.86 (0.89–3.91) *P*=.10
Embryology laboratory	0.63 (0.26–1.53) *P*=.30	2.31 (0.82–6.50) *P*=.11	1.01 (0.45–2.27) *P*=.97
Mandated state	1.35 (0.41–4.38) *P*=.62	0.8 (0.22–2.91) *P*=.75	0.46 (0.13–1.57) *P*=.27
Trainee clinic	1.34 (0.51–3.53) *P*=.55	0.68 (0.24–1.93) *P*=.47	-

*Note:* CI = confidence interval; IVF = in vitro fertilization; OR = odds ratio; REI = reproductive endocrinology and infertility

### Discounted Pricing and Lower-Cost IVF Approaches

**Supplemental Figure 3:**
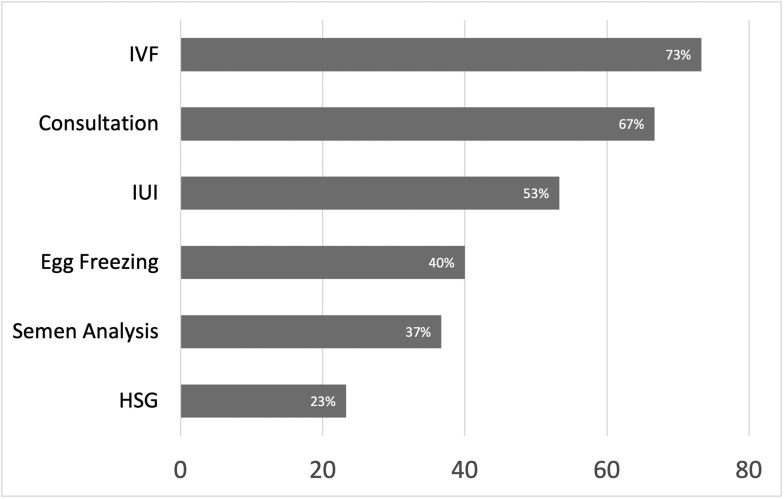
Discounted fertility services offered in reproductive endocrinology and infertility practices affiliated with obstetrics and gynecology residency programs (n = 30). HSG = hysterosalpingogram; IVF = in vitro fertilization; IUI = intrauterine insemination.

Of the 30 respondents offering discounted services at their primary site to expand access to infertility care, the most common method was discounted pricing for IVF (73%, n = 22), followed by discounting clinical consultation (70%, n = 21) and intrauterine insemination (53%, n = 16) (Supplemental Fig. 3). The provision of discounted prices for infertility services was positively correlated with the increasing practice size (odds ratio [OR], 2.29; 95% confidence interval [CI], 1.23–4.24; *P*=.01) and number of ART cycles performed annually (OR, 3.65; 95% CI, 1.48–9.02; *P*=.005). Academic REI practices trended to be more likely to have a lower-cost IVF program (OR, 3.6; 95% CI, 0.98–13.25; *P*=.05) (Table 3).

**Supplemental Figure 4:**
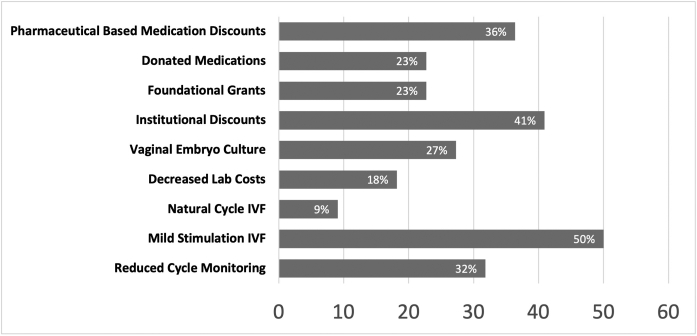
Approaches used to lower in vitro fertilization (IVF) cost in reproductive endocrinology and infertility practices affiliated with obstetrics and gynecology residency programs (n = 22).

For the clinics offering low-cost IVF (n = 22), the lower costs were achieved through the use of mild stimulation (50%, n = 11), fewer laboratory draws and/or ultrasounds during cycle monitoring (32%, n = 7), institutional-based discounts or write-offs (41%, n = 9), and pharmaceutical company-based medication discount programs (36%, n = 8) (Supplemental Fig. 4). Of practices with a low-cost IVF program, 40.9% (n = 9) were developed within the past 5 years. Low-cost IVF programs were staffed by a range of providers. Most (n = 20) were staffed by attendings, 6 by fellows, 3 by residents, and 3 by midlevel providers. Surprisingly, 64% (n = 14) were staffed by attendings alone, and 68% (n = 15) of low-cost IVF programs were reportedly not staffed by trainees.

### Reported Barriers

We asked participants to describe barriers to the provision or expansion of fertility services to patients who are medically underserved or unable to afford infertility care both in the main office for the practice and within the resident-staffed clinic, if one existed. Specifically, participants were asked 2 questions. First, “From your perspective, what barriers make it difficult to initiate or expand access to infertility care for patients who are medically underserved or unable to afford infertility care?” Regarding the main office practice, the most often cited barrier, by 84% of respondents (n = 67), was limited by insurance coverage. Participants quoted the difficulty in lowering treatment costs to an affordable range (76%, n = 61), lack of control of price structure (43%, n = 34), and negative effect of such clinical activities on profitability (21%, n = 17). The perceived financial barriers to expanding care did not differ significantly (*P* values of >.5) from practices in mandated states (n = 18) to those in nonmandated states (Supplemental Table 1).

Perceived patient-related barriers included patient health literacy (22%, n = 18), language barriers (16%, n = 13), and geographic distance to an IVF clinic (19%, n = 15). Logistic barriers included the lack of interest from administration (30%, n = 24), lack of knowledge of how to provide fertility care in lower-resource settings (20%, n = 16), bureaucracy encountered in starting new clinical programs (19%, n = 15), and lack of interest from clinicians in practice (18%, n = 14). Ethical barriers were cited less frequently; however, the most common was desire for equity for all patients (29%, n = 23). Additionally, 20% (n = 16) supported the idea that infertility services are considered elective and, thus, should not be offered in resource constrained settings or supported by public funding and the belief that resources should be directed to other more medically necessary clinical services (13%, n = 10).

**Figure 1:**
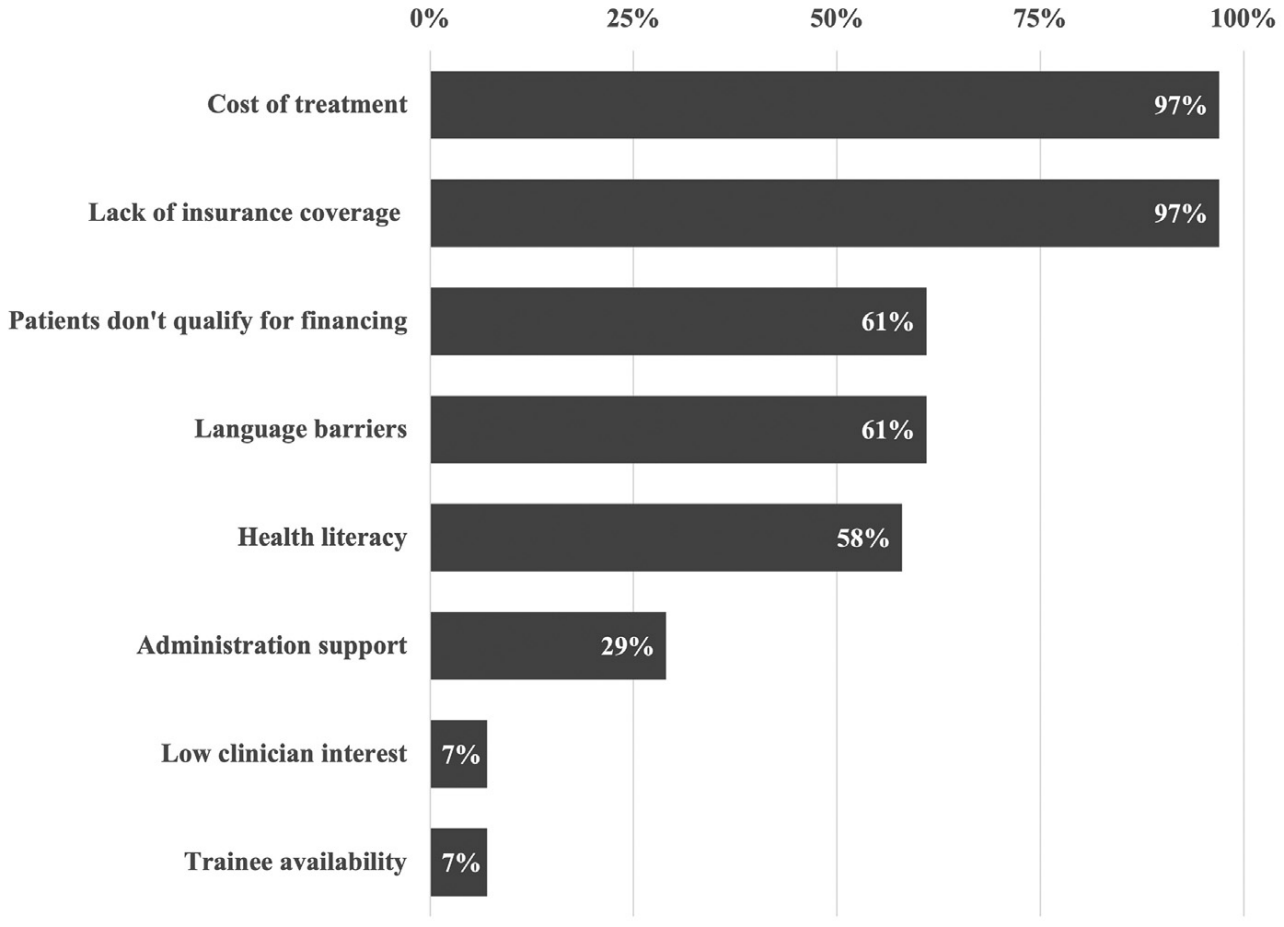
Reported barriers in the provision of infertility services to the underserved in obstetrics and gynecology trainee clinics (n = 31).

Regarding barriers in expanding services in the resident clinic, participants were asked “What barriers does your resident or fellow clinic encounter in providing fertility services to patients who are medically underserved or unable to afford infertility care?” Most respondents cited the prohibitive cost of treatment (97%, n = 30), lack of insurance or public health coverage (97%, n = 30), difficulty for patients with low income to qualify for loans or other financing plans (61%, n = 19), health literacy (61%, n = 19), and patient language (58%, n = 18) as barriers to expanding access to care. An additional percentage cited the low level of interest or support from hospital administration (29%, n = 9) or clinicians in the practice (7%, n = 2) or limited availability of trainees (7%, n = 2) (Fig. 1).

## Discussion

In September 2015, the ASRM hosted the Access to Care Summit in Washington, D.C., that resulted in publication of the White Paper (14). This document, a blueprint for the ASRM Access to Care Initiative, outlined 25 action items to address the wide disparities in access to infertility care that exist in the United States, and globally, with a goal for universal access to reproductive health care. These recommendations included the provision of unreimbursed care and donation of the clinical time and services to underserved, uninsured clinics. Furthermore, the utilization of simplified and lower-cost methods of treatment to reduce the cost burdens of infertility care was encouraged (15).

To our knowledge, our study is the first to broadly and systematically examine access to care initiatives in the setting of REI practices involved in the training of obstetrics and gynecology residents throughout the United States. Previously published research in this arena has been limited to programs at individual institutions or foundations to expand access to infertility care to the underserved (7, 11, 12, 16).

In our study, just over half (56%) of respondents, representing the full spectrum of the REI practice types associated with obstetrics and gynecology residency programs, reported at least one clinical access to care initiative to provide infertility care to patients who are underserved or unable to afford care. Approaches used by practices varied institutionally, but the most commonly used approaches involved the use of discounted treatment services, operating a low-cost IVF program, or having a resident- or fellow-staffed infertility clinic. The provision of discounted fertility services was correlated with the increasing practice size and number of ART cycles performed annually but not practice type (i.e., academic or private). This finding suggests that larger volume practices have greater capacity to be flexible on price structure than small practices. Academic REI practices were more likely to have a low-cost IVF program. For low-cost IVF programs, the lower costs were achieved in different ways through the use of mild stimulation, institutional-based discounts or write-offs, fewer laboratory draws and/or ultrasounds during cycle monitoring, and medication discount programs. Sites often utilized a combination of approaches to lower cost of care.

Less than half of obstetrics and gynecology residency programs (39%, n = 31) reported having an associated REI clinic, in which obstetrics and gynecology residents provide direct infertility care to populations who are medically underserved or unable to afford infertility services. This finding is of significance both in respect to an underutilized potential to expand outreach of infertility care to underserved communities and for optimizing REI training in obstetrics and gynecology residency. Residency programs are generally based in academic medical centers, community-based hospitals, and military medical facilities that traditionally have served broad demographics, including providing access to care to underserved communities and marginalized patient groups. Increasing utilization and engagement of trainees in clinical care can help lower cost barriers and improve access to care for patients who may not be able to afford or be able access services at other centers. Training institutions are often better equipped to provide care to patients with language and literacy barriers with greater availability and use of interpreters.

Studies have reported wide gaps and heterogeneity in resident education and clinical exposure to REI across obstetrics and gynecology residency programs in the United States (8, 17, 18). Increasing involvement of resident and fellow trainees in precepted REI care to the underserved is an opportunity for strengthening REI clinical training during obstetrics and gynecology residency. Enhanced exposure will likely increase competency and develop comfort among obstetrics and gynecology residents not only in the diagnostic evaluation and treatment of infertility but also in the full spectrum of REI clinical practice, including management of reproductive endocrine disorders. Graduates, some of whom will provide care in underserved areas without an available REI subspecialist, will be better prepared to provide care for their patients. Clinical care in a lower-resource, underserved setting provides both obstetrics and gynecology residents and REI fellows important exposure to infertility as it presents across diverse sociocultural and racial/ethnic demographics as well as disparities that exist. It also confers experience in navigating barriers in the provision of care. Furthermore, training in cost-effective clinical management, including simplified ovulation induction protocols and streamlined diagnostic evaluation, as well as the application of lower-cost treatments such as mild stimulation IVF, provides additional management tools for clinicians to expand options for their patients.

Recognizing and understanding barriers to accessing care are critical for reducing disparities to underserved and marginalized groups to improve both access and quality of care. Barriers reported by lower-income patients seeking infertility care identified affordability, insurance status, language and cultural barriers, communication, and bureaucracy within the public health system as challenges in accessing appropriate infertility care (7, 19). Additional barriers reported by patients included distance from clinic, required time off work, cultural values, language differences, social stigma, and fear (4, 7, 20, 21). Our survey examined barriers from the perspective of clinical providers in providing care or expanding access to care infertility to underserved patients. Respondents cited multiple barriers in providing low-cost infertility care including barriers around treatment costs, insurance coverage, profitability, and lack of institutional interest or support. They also identified patient health literacy and language barriers. Of note, perceived financial barriers to expanding care did not differ significantly in respondents from practices in mandated states to those in nonmandated states. This likely reflects that state insurance mandates apply to private insurance plans and not to local, state, or federal public health plans, underscoring that, even in states with comprehensive insurance mandates, significant disparities can still persist, particularly among low income and marginalized populations (22, 23, 24).

To our knowledge, our study is the first survey of access to care initiatives in REI practices associated with obstetrics and gynecology residency programs. Our findings fill a significant void in the literature on access to care initiatives and are important to better understanding the scope of the problem, challenges, and current practice to guide practice and policy changes as well as for the development of future studies. The strengths of our study include a comprehensive data collection of demographics, practice patterns, and perceived barriers among REI physicians who are educating obstetrics and gynecology residents in the United States. As there is no centrally maintained compilation of REI affiliations with obstetrics and gynecology residency programs, which includes academic, private, and military REI practices, this data set of REI resident education contacts in training programs had to be constructed for this study. Maintained and updated as a database, it could serve as a significant resource to improve REI resident education through facilitating dissemination of educational resources, curriculums, and utilization in future studies. While the response rate for our survey was not high at 30%, it is above the average 20% expected for a nonincentivized mailed survey and captured a demographically diverse set of data (25). Our respondents were distributed across diverse geographic regions, practice sizes, and clinical volume. That said, a selection bias may be present; the providers who responded to the survey are possibly more likely to be engaged in access to care than those who chose not to participate. It is conceivable that the percentage of REI clinics associated with obstetrics and gynecology residency programs that have practices to provide infertility care to the underserved may be an overestimate. Additionally, despite our broad survey of practices, the numbers of some of our outcome measures in the subanalysis are small and, thus, potentially subject to type II error.

## Conclusion

Our findings support that REI practices associated with obstetrics and gynecology residency programs utilize a diverse range of approaches to provide infertility care to the undeserved in the backdrop of considerable challenges and barriers. The findings underscore both wide gaps that exist and potential opportunities to improve infertility care to underserved patients and enhance the clinical training of residents and fellows.
