# Supplementary material for: Provision of infertility care for the underserved in reproductive endocrinology and infertility practices associated with obstetrics and gynecology residency training programs in the United States
Source: F S Rep. 2021 Nov 5;3(2 Suppl):106–13. doi: 10.1016/j.xfre.2021.11.002 (PMC9349242; doi:10.1016/j.xfre.2021.11.002)
Supplement: Supplementary file 1 — Supplemental Material [file mmc1.pdf]

Access to Care Survey of REI Divisions in OB/GYN Residency Programs

Access to infertility care is an on-going challenge in the field of reproductive endocrinology. We would like to learn more about your practice/division in order to survey what is currently being done in the community and help understand the challenges that practices face in expanding access to care. We would also like to know how OB/GYN resident and/or REI fellow trainees are involved in this care. Please take 5-10 minutes to complete the following survey. Thank you for your time and input!

1. Which best describes the structure of the Reproductive Endocrinology and Infertility (REI) practice at your institution?
Check all that apply.
- o Academic
- o Private practice
- o Hybrid academic/private practice
- o Military
- o Other: _________

2. In which state/district is your practice primarily based?
Mark only one circle.
- o Alabama
- o Alaska
- o Arizona
- o Arkansas
- o California
- o Colorado
- o Connecticut
- o Delaware
- o District of Columbia
- o Florida
- o Georgia
- o Hawaii
- o Idaho
- o Illinois
- o Indiana
- o Iowa
- o Kansas
- o Kentucky
- o Louisiana
- o Maine
- o Maryland
- o Massachusetts
- o Michigan
- o Minnesota

- Mississippi
- Missouri
- Montana
- Nebraska
- Nevada
- New Hampshire
- New Jersey
- New Mexico
- New York
- North Carolina
- North Dakota
- Ohio
- Oklahoma
- Oregon
- Pennsylvania
- Puerto Rico
- Rhode Island
- South Carolina
- South Dakota
- Tennessee
- Texas
- Utah
- Vermont
- Virginia
- Washington
- West Virginia
- Wisconsin
- Wyoming
- Other

3. How many REI specialist providers are there in your clinical practice, including fellowship-trained REI attending physicians and mid-level fertility-specialist providers (nurse practitioner or physician assistant)?

Mark only one.

- 0
- 1-2
- 3-5
- 6-8
- >8

4. How many TOTAL cumulative weeks do OB/GYN residents rotate through reproductive endocrinology & infertility during their residency? *
Mark only one circle.

- 0 weeks
- 1 weeks
- 2 weeks
- 3 weeks
- 4 weeks
- 5 weeks
- 6 weeks
- 7 week
- 8 weeks
- 9 weeks
- 10 weeks
- > 10 weeks
- I don't know
- Not applicable

5. How many OB/GYN residents graduate each year from your residency program?
Mark only one circle.

- 2
- 3
- 4
- 5
- 6
- 7
- 8
- 9
- 10
- 11
- 12
- >12
- I don't know
- Not applicable

6. Does your practice have an accredited fellowship in Reproductive Endocrinology and Infertility?
Mark only one circle.

- Yes
- No

7. Does your practice offer assisted reproductive technologies (ART) / in vitro fertilization (IVF)?
Mark only one circle.

- Yes, via an on-site embryology laboratory
- Yes, via an affiliate or contracted laboratory off-site
- No Skip to question 10.

8. How many assisted reproductive technology (ART) cycles did your practice perform in 2018? Please include all cycles started with the intent of oocyte retrieval, frozen embryo transfer and frozen egg thawing with subsequent embryo transfer (SART definition).
Mark only one circle.

- <200
- 201-500
- 501-1000
- >1000

9. Does your practice have a formalized oncofertility program with counseling and fertility preservation treatment for patients facing gonadotoxic treatment for cancer?
Mark only one circle.

- Yes
- No

10. In your estimation, approximately what percentage of patients in your practice cannot proceed with or prematurely discontinue infertility services due to lack of insurance or financial means?
Mark only one circle.

- <10%
- 10-25%
- 26-50%
- >50%
- I don't know

11. Does your practice offer fertility services at a discounted or lower price to patients who struggle to afford infertility care?
Mark only one circle.

- Yes Skip to question 12.
- No Skip to question 13.
- I don't know Skip to question 13.

Fertility Services

12. Which fertility services are offered at a discounted or lower price to patients who are unable to afford infertility care?
Check all that apply.

- Consultation
- Hysterosalpingogram
- Semen analysis
- Clomiphene, letrozole or gonadotropin-based IUI
- Oocyte cryopreservation (Egg freezing)
- In vitro fertilization (IVF)

- Other: ____________
- Lower cost & complexity IVF

13. Does your practice have a lower cost and/or lower complexity IVF option or program developed to improve access of care for lower income patients?
Mark only one circle.
- Yes
- No Skip to question 19.
- I don't know Skip to question 19.

14. For approximately how many years has your practice's low cost and/or complexity IVF program been offered?
Mark only one circle.
- < 1 year
- 1 year
- 2-5 years
- 6-10 years
- >10 years
- I don't know

15. On average, approximately how many cycles of low cost and/or complexity IVF are undertaken each year?
Mark only one circle.
- <10
- 11-20
- 21-30
- 31-40
- 41-50
- >50
- I don't know

16. What level of clinical providers primarily see patients in this lower cost and/or complexity IVF program?
Check all that apply.
- OB/GYN Residents
- REI Fellows
- REI attendings only
- Mid-level providers (nurse midwives, nurse practitioners, physician assistants)
- Other:_______________

17. How does your practice achieve a lower cost of IVF for patients in this program?
Check all that apply.
- Reduced cycle monitoring (fewer lab draws and/or ultrasounds)
- Mild stimulation IVF (use of lower doses of gonadtropin medication)

- Natural cycle IVF
- Decreased use of anesthesia (e.g. use of premedication rather than conscious sedation)
- Decreased laboratory handling of gametes and embryos (e.g. no ICSI, assisted hatching, no extended culture)
- Vaginal embryo culture/incubation (i.e.: INVOcell)
- Involvement of trainees (e.g. fellows or residents) in clinical care
- Use of mid-level providers (nurse midwives, nurse practitioners, physician assistants)
- Institutional discounts or write-off of costs
- Foundational grants
- Donated medications from other patients
- Pharmaceutical company-based medication discount programs
- Other:_______________

18. How do patients qualify for inclusion in this program?
Check all that apply.

- Direct referral from clinics (e.g. REI or Gynecology) within your institution
- Financial criteria; self-reported by patients without validation
- Financial criteria; validated by tax records, pay-stubs or other instruments
- No eligibility criteria requirements for patients
- Other:_______________

Trainee REI Curriculum

19. Does the OB/GYN residency program and/or fellowship program have a structured educational curriculum on any of the following: CHECK ALL THAT APPLY *
Check all that apply.

- Disparities and barriers in access to infertility care
- Experience of infertility across different sociocultural demographics and underserved communities, such as in immigrant populations
- Clinical management of infertility in lower resource settings
- Infertility in a global health context
- None of the above
- Other:_______________

Resident/Fellow Clinic

20. Does your practice have an infertility clinic staffed with resident or fellow trainee physicians to provide care to patients who are medically underserved or unable to afford infertility services? For example, a OB/GYN resident operated REI-clinic at a county hospital. *
Mark only one circle.

- Yes
- No Skip to question 26.
- I don't know Skip to question 26.

21. How is your resident/fellow clinic staffed?
Mark only one circle.

- Resident-only clinic with GYN or REI attending
- Resident-only clinic with an REI fellow serving as attending
- Fellow-only clinic with REI attending
- Combined resident and fellow clinic with REI attending
- Other:_______________

22. Where is this clinic held?
Mark only one circle.

- County or public medical center
- Comprehensive IVF center
- Resident gynecology clinic
- Other:_______________

23. How often is this resident and/or fellow clinic offered?
Mark only one circle.

- Daily
- Weekly
- Twice monthly
- Monthly
- Other:_______________

24. Which fertility services are directly provided (i.e. performed in house and not referred out) to patients in the resident and/or fellow clinics?
Check all that apply.

- Consultation
- Hysterosalpingogram (HSG)
- Saline-infusion sonography
- Pelvic ultrasound
- Semen analysis
- Clomiphene/Letrozole based ovulation induction / superovulation
- Gonadotrophin based ovulation induction / superovulation
- In-clinic sperm preparation for intrauterine insemination (IUI)
- Intrauterine insemination (IUI)
- Oocyte cryopreservation
- In vitro fertilization
- Vaginal embryo culture/incubation (i.e.: INVOcell)
- Donor egg IVF
- Reversal surgery after tubal ligation
- Laparoscopy for tubal adhesive disease
- Tubal recanalization
- Assisted conception for HIV serodiscordant couples

- o Diagnostic evaluation and management of endocrinopathies (e.g. PCOS) in patients not actively
- o desiring pregnancy
- o Management of endometriosis-associated pelvic pain in patients not actively desiring pregnancy
- o Diagnostic evaluation and management of congenital uterine anomalies and disorders of sexual
- o development
- o Other:_______________

Barriers to Providing Care

25. What barriers does your resident or fellow clinic encounter in providing fertility services to patients who are medically underserved or unable to afford infertility care?
Check all that apply.

- o Prohibitive cost of treatment/services to lower income patients
- o Limited or lack of insurance or federal/state health coverage
- o Difficulty in patients with low income to qualify for loans or other financing plans
- o Patient language barriers
- o Patient health literacy (i.e. understanding of disease and treatment options)
- o Limited of lack of interest / support from practice/ hospital administration
- o Limited or lack of Interest / support of clinician providers
- o Limited availability of resident and/or fellow clinical coverage
- o None of the above
- o I am unsure
- o Other:_______________

Infertility Care in Global Settings

26. In your program, do OB/GYN residents and/or REI fellows get clinical exposure to infertility care for medically underserved populations internationally? *
Mark only one circle.

- o Yes
- o No Skip to question 28.
- o I don't know Skip to question 28.

27. In what ways do OB/GYN residents and/or REI fellows in your program get clinical exposure to infertility care for medically underserved populations internationally?
Check all that apply.

- o Health policy work
- o Public health campaigns or community outreach

- Capacity building with local in-country partners and health care organizations
- Research
- Direct provision of clinical care through consultation and diagnostic infertility evaluation
- Direct provision of clinical care through surgical treatment (e.g. laparoscopy for tubal disease)
- Direct provision of clinical care through clomiphene/letrozole based ovulation induction/superovulation
- Direct provision of clinical care through gonadotropin based ovulation induction/superovulation
- Direct provision of clinical care through intrauterine insemination
- Direct provision of clinical care through in vitro fertilization
- Direct provision of clinical care through working with the IVF embryology lab
- Direct provision of clinical care through assisting safe conception for HIV serodiscordant couples
- Other:
- Infertility Care in Local Settings

28. From your perspective, what barriers make it difficult for academic programs to initiate or expand access to infertility care for patients in local communities who are medically underserved or unable to afford infertility care?
Check all that apply.

- Difficulty in lowering treatment costs to an affordable range that would expand access to care
- Limited / lack of insurance or public health coverage for infertility diagnostics and treatment
- Lack of control of price structure
- Desire for equity in servicing all patients, for example: not offering differing charges for the same treatment
- Concern that patients may misrepresent their financial situation to take advantage of systems offered
- Concern for practice image or perception by other patients
- Unsure of how to access these populations
- Patient language barriers
- Patient health literacy (i.e. understanding of disease and treatment options)
- Patient geographic distance to the IVF clinic
- Lack of interest / support from department / hospital administration
- Bureaucracy and inertia encountered in starting new clinical programs
- Lack of interest / support from clinicians in practice
- Lack of knowledge of how to provide fertility care in lower resource settings
- Lack of knowledge of how to provide fertility care to infertility patients from different sociocultural
- backgrounds
- Concern that initiatives designed to increasing access of care might be perceived as being driven by motives of financial gain to increase clinic revenue

- Concern for negative effect of such clinical activities on clinic's profitability and bottom line
- Concern for the ability for lower income patients to financially support cost of raising a child
- Belief that infertility services should be considered elective and thus not offered in resource constrained settings or supported by public funding
- Belief that hospital or public health resources should be directed to other more medically-necessary clinical services
- None of the above
- I am unsure
- Other:_______________
