# Supplementary material for: Provision of infertility care for the underserved in reproductive endocrinology and infertility practices associated with obstetrics and gynecology residency training programs in the United States
Source: F S Rep. 2021 Nov 5;3(2 Suppl):106–13. doi: 10.1016/j.xfre.2021.11.002 (PMC9349242; doi:10.1016/j.xfre.2021.11.002)
Supplement: Supplemental Table 1 [file mmc2.docx]

**Supplemental Table 1: Reported barriers to expanding care in insurance mandated vs. nonmandated states**

|  | **Overall (n=80)** | **Mandated**  **(n=18)** | **Non-mandated (n=62)** | **Mandated vs. non-mandated**  ***P*-value** |
| --- | --- | --- | --- | --- |
| **Insurance coverage** | 81%  (n=65) | 83%  (n=15) | 78%  (n=48) | 0.751* |
| **Lowering treatment costs to an affordable range** | 77%  (n=62) | 72%  (n=13) | 80%  (n=50) | 0.577* |
| **Lack of control of price structure** | 43%  (n=34) | 50%  (n=9) | 40%  (n=25) | 0.596**^~^** |
| **Concern for negative effect profitability** | 21%  (n=17) | 11%  (n=2) | 23 %  (n=15) | 0.503**^~^** |

*χ^2^~fisher’s exact
